# Performance evaluation of the Asante Rapid Recency Assay for verification of HIV diagnosis and detection of recent HIV-1 infections: Implications for epidemic control

**DOI:** 10.1371/journal.pgph.0000316

**Published:** 2022-05-03

**Authors:** Ernest L. Yufenyuy, Mervi Detorio, Trudy Dobbs, Hetal K. Patel, Keisha Jackson, Shanmugam Vedapuri, Bharat S. Parekh

**Affiliations:** Division of Global HIV and TB, Center for Global Health, Centers for Disease Control and Prevention, Atlanta, Georgia, United States of America; University of California Irvine, UNITED STATES

## Abstract

We previously described development of a rapid test for recent infection (RTRI) that can diagnose HIV infection and detect HIV-1 recent infections in a single device. This technology was transferred to a commercial partner as Asante Rapid Recency Assay (ARRA). We evaluated performance of the ARRA kits in the laboratory using a well-characterized panel of specimens. The plasma specimen panel (N = 1500) included HIV-1 (N = 570), HIV-2 (N = 10), and HIV-negatives (N = 920) representing multiple subtypes and geographic locations. Reference diagnostic data were generated using the Bio-Rad HIV-1-2-O EIA/Western blot algorithm with further serotyping performed using the Multispot HIV-1/2 assay. The LAg-Avidity EIA was used to generate reference data on recent and long-term infection for HIV-1 positive specimens at a normalized optical density (ODn) cutoff of 2.0 corresponding to a mean duration of about 6 months. All specimens were tested with ARRA according to the manufacturer’s recommendations. Test strips were also read for line intensities using a reader and results were correlated with visual interpretation. ARRA’s positive verification line (PVL) correctly classified 575 of 580 HIV-positive and 910 of 920 negative specimens resulting in a sensitivity of 99.1% (95% CI: 98.0–99.6) and specificity of 98.9% (95% CI: 98.1–99.4), respectively. The reader-based classification was similar for PVL with sensitivity of 99.3% (576/580) and specificity of 98.8% (909/920). ARRA’s long-term line (LTL) classified 109 of 565 HIV-1 specimens as recent and 456 as long-term compared to 98 as recent and 467 as long-term (LT) by LAg-Avidity EIA (cutoff ODn = 2.0), suggesting a mean duration of recent infection (MDRI) close to 6 months. Agreement of ARRA with LAg recent cases was 81.6% (80/98) and LT cases was 93.8% (438/467), with an overall agreement of 91.7% (kappa = 0.72). The reader (cutoff 2.9) classified 109/566 specimens as recent infections compared to 99 by the LAg-Avidity EIA for recency agreement of 81.8% (81/99), LT agreement of 9% (439/467) with overall agreement of 91.9% (kappa = 0.72). The agreement between visual interpretation and strip reader was 99.9% (95% CI: 99.6–99.9) for the PVL and 98.1% (95% CI: 96.6–98.9) for the LTL. ARRA performed well with HIV diagnostic sensitivity >99% and specificity >98%. Its ability to identify recent infections is comparable to the LA-Avidity EIA corresponding to an MDRI of about 6 months. This point-of-care assay has implications for real-time surveillance of new infections among newly diagnosed individuals for targeted prevention and interrupting ongoing transmission thus accelerating epidemic control.

## Introduction

The HIV pandemic has been with us for more than 40 years and global HIV burden is now estimated at about 38 million people living with HIV [1]. However, with major investments and commitment from national and international partners, including Ministries of Health, most countries have made major strides in changing the trajectory of the epidemic in most populations and countries in the last 15–20 years. Globally, we have scaled up HIV testing, accelerated prevention of mother-to-child transmission, developed new and effective treatments, placed millions of people on antiretroviral treatment (ART) and innovated novel prevention approaches, resulting in significant reduction in estimated new infections from 2.7 million in 2000 to 1.7 million in 2019 [1]. However, the progress is varied with some countries reaching UNAIDS 90-90-90 goals while others are still working to achieve these goals [2].

Identifying ongoing HIV transmission and targeting prevention strategies for populations with high incidence are important for accelerating epidemic control [3, 4]. This can be best achieved by using tests that can detect recently infected individuals [5–8]. Considerable efforts and resources have been devoted to the development of laboratory assays to detect recent HIV-1 infections [9–23]. However, only two of these assays, both developed in our laboratory, have been commercialized for specific application to estimate HIV-1 incidence in cross-sectional population: the BED-Capture Enzyme Immunoassay (BED-CEIA) [24] and the Limiting Antigen (LAg) Avidity EIA [17]. The LAg-Avidity EIA was developed and commercialized in 2012 to address some of the limitations of the BED assay and is widely used in many countries worldwide [17, 25–34]. Most recently, the LAg-Avidity EIA has been used in population-based HIV impact assessment (PHIA) surveys as a key methodology to estimate incidence in several countries [35, 36]. The LAg-Avidity EIA uses a multi-subtype gp41 protein, rIDR-M, covering the immunodominant region of gp41 of HIV-1, which permits equivalent detection of antibody avidity from all major subtypes [20]. The assay is based on a novel approach of using limiting amount of antigen thereby limiting the ability of low avidity antibodies (usually from recent infection) to bind, while allowing high avidity antibodies present during long term infections to bind [17, 20].

Until recently, the utility of incidence assays was limited to the estimation of HIV incidence from cross-sectional population surveys, which may also permit association of risk factors and identification of populations with high incidence. The utility of incidence assays has evolved to include the identification of recently infected individuals in some European countries for contact tracing and index testing [37, 38]. However, such application of laboratory-based tests for recent infection have been limited due to the delay in results since these assays are conducted in a central laboratory and results may not be available for several days or weeks. Development of a rapid test for recent infection allows one to perform HIV recency testing in the routine HIV program in real time and utilize data for targeted prevention [18]. Our laboratory developed a rapid test for recent infection [18] which now has been commercialized by Sedia Biosciences (Portland, OR) as Asante Rapid Recency Assay (ARRA).

The ARRA is a strip-test that combines HIV diagnosis with marker of time since infection as previously described [18]. This is achieved by including an additional line where antigen is striped at limiting antigen concentration to distinguish recent from long term infection. The principle of the assay is similar to the laboratory-based LAg-Avidity EIA and uses the same rIDR-M antigen but in a rapid test format. The lines can be read visually as well as with a strip reader, which produces quantitative results in intensity units (IU). However, ARRA has been not evaluated using a large panel of specimens that mimic cross-sectional specimens. We describe here the performance evaluation of this test kit using a large well-characterized global panel of specimens. The purpose of this evaluation was to compare the performance of both the PVL and the LTL on the Asante strips to the standard HIV diagnostic algorithm and the LAg-Avidity EIA for recency classification, respectively.

## Methods

### Specimens

Our world-wide panel of plasma specimens (N = 1500; HIV-1 = 570, HIV-2 = 10, Neg = 920) was derived from rejected blood units from adult donors in blood banks from Kenya, Uganda, Cameroon, Cote d’Ivoire, Sierra Leone, South Africa, Thailand, and the United States, collected in 2005–2006 for validation of HIV rapid tests in support of President’s Emergency Plan for AIDS Relief (PEPFAR) under a CDC approved protocol [39]. Blood units were rejected due their reactivity to one or more pathogens (HIV, Syphilis, Hepatitis, etc.) that could be potentially transmitted by blood transfusion. The protocol was approved by the CDC as non-research activity and did not require full IRB review due to use of unlinked blood donor specimens for test evaluations. Informed consent was obtained from the donors for use of their specimens for quality assurance and test evaluations. De-identified blood units were separated into plasma at site, frozen at -20± C, and transported by air to our laboratory on dry ice. Upon receipt, unlinked specimen IDs were matched with specimen log, given a new CDC ID, and aliquoted for long-term storage at -70± C. The specimens were characterized for the presence or absence of HIV antibodies using the 3^rd^ generation Genetic Systems Enzyme Immunoassay (EIA) (Bio-Rad Laboratory, Hercules, CA) followed by Cambridge Biotech HIV-1 Western blot assay (Maxim, Rockville, MD) for confirmation. HIV-1 and HIV-2 serotyping was done using the Multispot HIV-1/2 (Bio-Rad) assay. The HIV-positive specimens were inherently made up of different subtypes and recombinants due to diverse geographic origins, although sequencing was not performed. Additional characterization of HIV-1 positive specimens was done using the LAg-Avidity EIA (Sedia Biosciences, Portland, OR) to classify specimens as either recent or long-term infections. The specimens were not tested for anti-retroviral (ARV) drugs but were likely from ARV naïve individuals because they were collected from blood donors in 2005–2006 when treatment coverage was low in most countries in sub-Saharan Africa. The panel consisted of HIV positive (n = 580; of which 570 were HIV-1 and 10 were HIV-2), and HIV negative specimens (n = 920). The HIV-1 specimens (n = 570) included 103 (18.1%) recent and 467 (81.9%) long-term infections as classified by the LAg-Avidity EIA at the threshold cutoff of 2.0 ODn, matching the MDRI of about 6 months [25]. No other clinical information was available for individual donors.

### Assay procedure

The ARRA was performed according to the manufacturer’s instructions [40]. Briefly, serum or plasma specimens were brought to room temperature and approximately 5μl of sample was collected using the specimen collection loop provided in the kit and transferred into a buffer tube containing 0.5mL of buffer and mixed. After discarding the specimen collection loop, a test strip was dropped into the tube and incubated for 20 minutes. Developed test strips were removed after 20 min, dabbed onto filter paper to remove excess buffer and then read visually as recent, long-term, or HIV negative followed by reading with Asante strip reader by the same technician (see below). Visual interpretation was done first to avoid any bias that may result from reader-based interpretation. The results were recorded in a worksheet, transferred to a central database and analyzed (see Data and Statistical Analysis sections below).

### Interpretation of results

**Fig 1:**
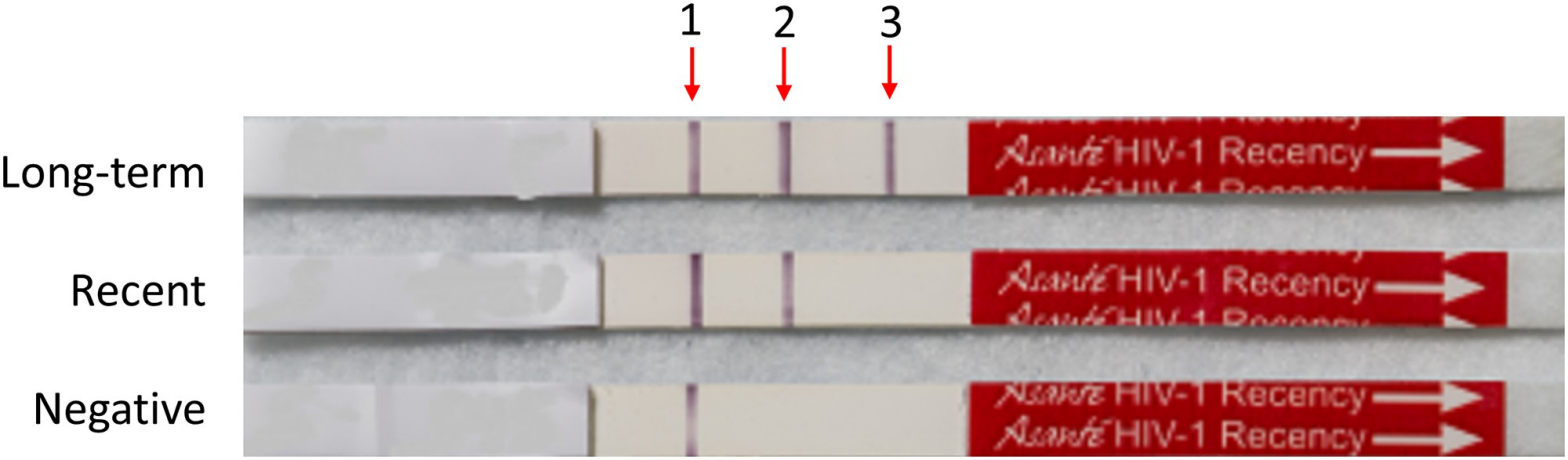
Interpretation of the Asante Rapid Recency Assay results is based on the presence or absence of specific lines. Arrows 1, 2 and 3 represent control line (CL), positive verification line (PVL) and long-term line (LTL), respectively. Presence of all three lines CL, PVL and LTL indicate HIV-positive diagnosis with long-term infection. The presence of CL and PVL indicate HIV-positive diagnosis with recent infection while the presence of only the control line (CL) indicates an HIV-seronegative sample. Quantitative results (in intensity units) can also be obtained with a strip reader.

Visually, the presence of all three lines [control line (CL), positive verification line (PVL), and long-term line (LTL)] indicated HIV-positive specimen with long term infection; presence of only two lines (CL and PVL) indicated HIV-positive specimens with recent infection; and presence of only CL indicated a seronegative specimen (Fig 1). The Asante strip reader measures the intensity of the three lines whose values are read in intensity units (IU). The reader cutoff for the PVL was 2.8 IU, where IU≥2.8 were HIV-reactive/positive and IU<2.8 were HIV negative. For the separation of recent from long term infections we used a cutoff of 2.9 IU for the LTL because it matched well with visual results (see Results section). IU of <2.9 on HIV-1 positive specimen were classified as recent infection, while IU≥2.9 were long term.

### Data analysis

The performance of the PVL was assessed by calculating the sensitivity and specificity of the PVL with reference to the standard algorithm: EIA + Western Blot, and Multispot or Geenius for serotyping. The performance of the LTL was calculated by correlating results from the LAg-Avidity EIA and Asante LTL for specimens with HIV-1 infections. Asante visual and Asante strip reader results were also compared.

### Statistical analysis

This was performed using both Microsoft Excel (See S1 Data) and GraphPad Prism. The sensitivity and specificity of the assay for PVL were calculated using numerical values of true positives (TP), false positives (FP), true negatives (TN) and false negatives (FN). Agreement between the standard diagnostic algorithm and the ARRA were assessed using *Kappa* values where *Kappa* values were categorized as good (0.61 to 0.8), very good (0.81 to 0.99) and perfect (0.99 to 1.0). Two-by-two table and *Kappa* values were calculated for LTL comparing rapid test for recent infection (RTRI) classification of recent or LT to classification by LAg-Avidity EIA. The Spearman ranked correlation coefficient was calculated for the ODn of the LAg-Avidity EIA and the reader values of ARRA LTL. Percent agreement was calculated between Asante visual and Asante strip reader results, between visual and standard assays, and between strip reader and standard assays; all with two-sided 95% Wilson score confidence intervals.

## Results

### Sensitivity and specificity of the positive verification line (PVL)

Of the 580 HIV positive specimens in the panel, the ARRA correctly identified 575 as HIV positive with 5 false negatives, with visual interpretation giving a sensitivity of 99.1% (95% CI: 98.0%-99.6%) (Table 1A). The assay also correctly identified 910 of 920 negative specimens; 10 specimens were false-positive resulting in a specificity of 98.9% (98.0%-99.4%). The reader-based interpretation using a manufacturer recommended cutoff of 2.8 was similar to visual interpretation, yielding a sensitivity and specificity of 99.3% (576/580) and 98.8% (909/920), respectively (Table 1B). In both cases, *kappa* statistics comparing reference results with visual or reader-based interpretation were almost perfect (0.979) [41].

**Table 1:** Two-by-two tables comparing EIA/Western Blot based final HIV diagnostic results with A) visual interpretation for the positive verification line and B) hand-held reader-based interpretation of the positive verification line of the Asante Recency Assay, N = 1500. Sensitivity, specificity, % accuracy and kappa values are shown (with 95% confidence intervals).

**A**				
		EIA/WB Algorithm		
Asante PVL (Visual)		HIV-positive	HIV-negative	Total
	HIV-positive	575	10	585
	HIV-negative	5	910	915
	Total	580	920	1500
**B**				
		EIA/WB Algorithm		
Asante PVL (Reader @2.8 IU)		HIV-positive	HIV-negative	Total
	HIV-positive	576	11	585
	HIV-negative	4	909	915
	Total	580	920	1500

A: Sensitivity = 99.1% (98.0–99.6), Specificity = 98.9% (98.0–99.4%), % Agreement = 99.0% (98.4–99.4), Kappa = 0.979 (0.968–0.990)

B: Sensitivity = 99.3% (98.2–99.7), Specificity = 98.8% (97.9–99.3%). % Agreement = 99.0% (98.4–99.4), Kappa = 0.979 (0.968–0.990)

### Detection of recent or Long-term (LT) infections

The performance of the ARRA LTL was assessed by comparing the LTL visual results with the LAg-Avidity EIA at an ODn cutoff of 2.0, matching with the mean duration of recent infection (MDRI) of about 6 months [25]. The LAg-Avidity-EIA classified 98 of the qualified 566 HIV-1 specimens as recent and the ARRA classified 109 as recent (Table 2A); 4 ARRA false-negatives were excluded from the analysis. Eighty (81.6%) of 98 LAg recent cases were recent and 438 (93.8%) of 467 LAg LT cases were LT by the ARRA, yielding an overall agreement of 91.7% (518/565) and kappa of 0.722. For reader-based interpretation, a cutoff of 3.0 IU recommended by the manufacturer was initially also used for analysis, but the LAg-Avidity EIA better correlated to the Asante strip reader at a cutoff of 2.9 IU which also correlated best with visual results (see below, Fig 3). Using this cutoff for LTL, results matched well with LAg-EIA (Table 2B) and were similar to visual interpretation, with an overall agreement of 91.9% and kappa of 0.729. The agreement between visual and reader-based interpretation for classification of recent or LT infection is shown in Table 2C demonstrating high level of overall agreement (>98%) and almost perfect kappa (0.937).

**Table 2:** Two-by- two tables comparing Asante Rapid Recency Assay results with LAg-Avidity EIA results for classification of recent or LT infections using (A) visual interpretation and B) strip reader @ cutoff of 2.9. C) Comparison of visual results with those generated with the strip reader for recency classification. Percent agreement and kappa values are shown for each comparison (with 95% CI).

**A**				
		LAg-Avidity EIA		
Asante LTL (Visual)		Recent	Long-Term	Total
	Recent	80	29	109
	Long-Term	18	438	456
	Total	98	467	565
**B**				
		LAg-Avidity EIA		
Asante LTL (Reader @2.9 IU)		Recent	Long-Term	Total
	Recent	81	28	109
	Long-Term	18	439	457
	Total	99	467	566
**C**				
		Visual Classification		
Reader Interpretation @2.9 IU)		Recent	Long-Term	Total
	Recent	103	6	109
	Long-Term	5	451	456
	Total	108	457	565

A: Recency Agreement = 81.6%, Long-Term Agreement = 93.8%, Overall % Agreement = 91.7% (89.1–93.7), Kappa = 0.722 (0.648–0.797)

B: Recency Agreement = 81.8%, Long-Term Agreement = 94%, Overall % Agreement = 91.9% (89.3–93.9), Kappa = 0.722 (0.648–0.797)

C: Recency Agreement = 95.4%, Long-Term Agreement = 98.7%, Overall % Agreement = 98.1% (86.6–98.9), Kappa = 0.937 (0.901–0.974)

**Fig 2:**
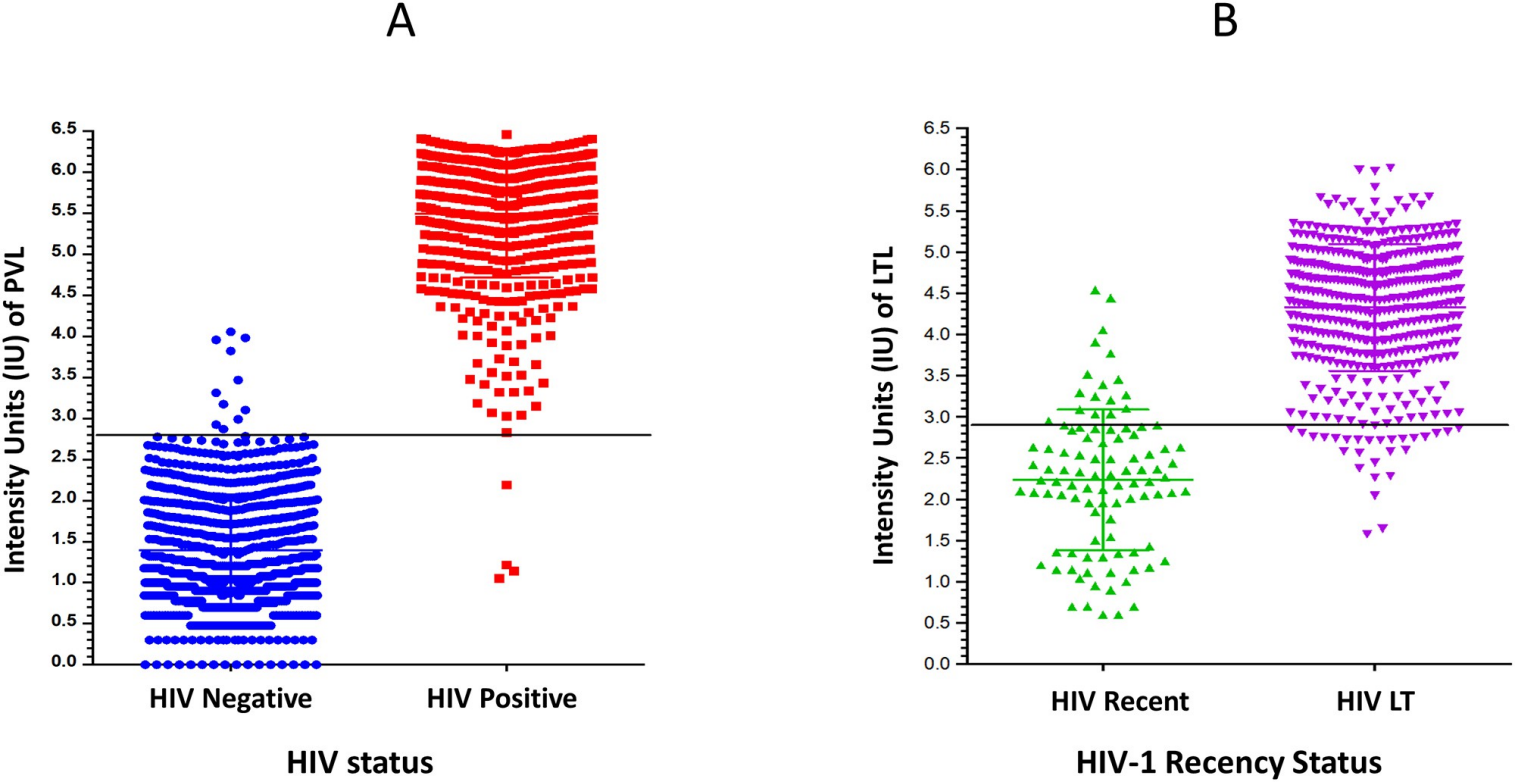
Distribution of reader values in IU for (A) positive verification line (PVL) for HIV-positive and negative specimens and B) long-term line (LTL) for HIV-1 recent and long-term specimens as classified by LAg-Avidity EIA. Horizontal lines represent threshold cutoff values of 2.8 (PVL) and 2.9 (LTL).

We further examined quantitative reader values for HIV diagnosis (PVL) or recency status (LTL). Distribution of the Asante reader values for the PVL for HIV-positive and negative specimens is depicted in Fig 2A showing overall concordance for HIV diagnosis and reader values for a few false positives (N = 11) and false negatives (N = 4). Similarly, distribution of the Asante reader values for the LTL for HIV-1 recent and LT specimens, as classified by LAg-Avidity EIA, is shown in Fig 2B demonstrating concordance and switches among recent and LT infections when classified by ARRA compared to LAg EIA.

### Comparison of visual reading with Asante strip reader

**Fig 3:**
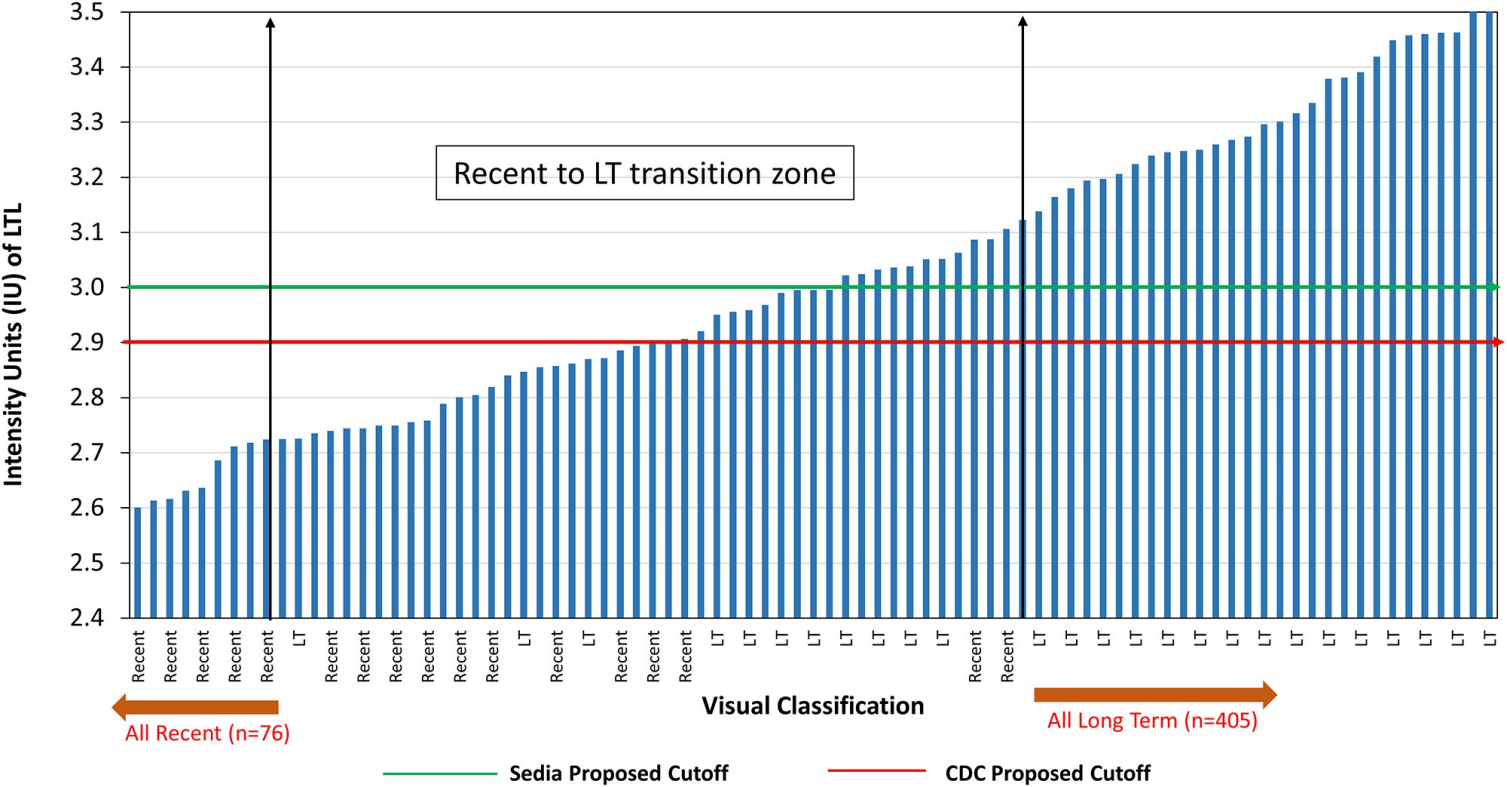
Graphical correlation of visual and strip reader IU values. The results show strip reader values arranged in ascending order and the corresponding visual result. Shown is a transition zone between two vertical arrows where visual representation and strip reader results may not agree. As shown, all values are close to the cutoff and range between >2.7 IU to <3.2 IU. Horizontal line at 3.0 IU is a manufacturer-suggested cutoff, while line at 2.9 is the cutoff that matches well with visual interpretation.

The percentage agreement between Asante visual and Asante strip reader in the interpretation of the PVL was 99.87% (95% CI: 99.52–99.98) with a *kappa* value of 0.997 (95% CI: 99.3–100). The percent agreement was 98.05% (95% CI: 96.54–99.02) for the LTL with a *kappa* value of 0.937 (0.901–0.974) (Table 2C). Specimens that had discrepant classification on the LTL were primarily located close to the cutoff of 2.9 and ranged from 2.7< to <3.2. Specimens in this zone were referred to as grey zone specimens and could occasionally switch between recent and long term. Fig 3 represents the relationship between visual interpretation and reader values with specimens arranged in ascending order of LT IU values. Visual results matched better with reader interpretation when cutoff of 2.9 was used for the LTL instead of cutoff of 3.0 as recommended by the manufacturer.

## Discussion

Access to HIV diagnostic testing has greatly expanded globally due to availability of point-of-care (POC) rapid diagnostic tests that are simple, inexpensive, and easy to perform. However, use of assays to detect recent HIV-1 infections has been limited mainly to cross-sectional surveys for estimation of incidence. Identification of recent infection is critical for epidemic control since evidence suggest that a high proportion of new infections originate from recently infected individuals [42–45]. However, identifying recent infections at POC has been difficult because of the complexities associated with current laboratory-based incidence assays that require collection of the specimens, transportation, processing, and storage before testing can be conducted at a centralized facility. Moreover, most HIV-1 incidence tests are technically complex, take several hours to perform, may require sophisticated equipment and specimen transport if testing will be done in the central laboratory [17, 46].

Our laboratory validation of the ARRA, a POC test that can simultaneously confirm HIV diagnosis and identify recent from long-term infections, shows great promise. This is the first evaluation of Asante Rapid Recency Assay to assess performance of both PV line and LT line. The assay showed great performance for HIV diagnosis with high sensitivity and specificity (99.1% and 98.9%, respectively; Table 1A) meeting and surpassing WHO-PQ/FDA requirements of minimum sensitivity of 99% or greater and specificity of 98% or greater [47]. In addition, ARRA simultaneously identified recent and LT infections in our cross-sectional specimen set with results similar to the LAg-Avidity EIA (>91% concordance and kappa >0.72) (Table 2A). The discordant rate of about 8% for detection of recent/LT cases between the two methodologies is not unexpected considering several cases are close to the cutoff (Fig 3B) and can switch between recent and LT status. This should have minimal impact on intended use of the assay for recent infection surveillance in programmatic settings and identifying risk factors of acquiring new infections for targeted prevention. Performance among subtype B specimens (n = 35) from the U.S. show 94.3% agreement with the LAg-EIA and non-B subtype specimens (n = 535) from other countries show 90.7% agreement with the LAg-EIA. However, only 35/570 (6.1%) HIV-1 specimens were from the U.S, making this difference is not statistically meaningful.

Combining HIV diagnostic testing with recent infection detection in a single test is important because often HIV false-positive results have been reported in the surveys and in the HIV program [48–50]. The testing of false-positive specimens with recent infection assays, without the diagnosis component, can result in misinterpretation since they will be misclassified as recent. Therefore, the laboratory-based LAg-Avidity EIA which only includes recency testing requires further confirmation of HIV diagnosis of individuals when ODn<0.4 to rule out false positives [40]. The ARRA that includes verification of HIV diagnosis serves to confirm HIV-positive status at the same time while classifying individuals as recent or LT.

Validation of antibody-based recency assays can be conducted by 1) use of longitudinal specimens (seroconversion panels) from recently infected persons to confirm expected antibody maturation profile and to determine the MDRI, 2) using specimens from persons with long-term infections (>1 year) to determine the proportion likely to be misclassified as false-recent and understand the reasons (e.g. elite controllers, ART) for misclassification for potential mitigation (e.g. viral load), and 3) comparison with a previously validated test (such as LAg-Avidity EIA) using a cross-sectional specimen set. We describe here the latter approach that allowed not only comparative evaluation of ARRA but also helped infer the MDRI without the use of seroconversion panels that are increasingly difficult to get.

The MDRI of the ARRA was inferred from correlation with the LAg-Avidity EIA data. An ODn cutoff of 2.0 correlated well with the long-term line on the ARRA (Table 2 and Fig 2), from which we inferred a tentative MDRI for the ARRA of approximately 6 months [25]. Additional work is underway to confirm the MDRI of the ARRA using a series of seroconversion panels. However, the MDRI is important for estimating HIV-1 incidence in a population, which is not the intended use of the assay at POC. ARRA is designed to identify recently infected individuals in routine HIV testing services, as part of surveillance of recent infections in HIV program for targeted prevention. Moreover, subtype differences will have minimal influence on the classification of recent and long-term infections which is interpreted as less than or greater than 1 year respectively, when interpreted at the individual level. There is a distribution around MDRI indicating individual may transition from recent to LT status earlier or later than 6 months, but almost all will transition within 1 year. Identifying individuals during this early phase can help interrupt ongoing transmission during their lifetime.

Until recently the utility of incidence testing was mostly limited to the estimation of HIV incidence at the population level, except for a few cases in the developed world where it has been used for case-based surveillance [38]. At the population level, cross-sectional national surveys have been conducted periodically (3–5 years) and incidence estimates from these surveys in sub-Saharan Africa have helped in the allocation of resources, defining prevention programs, and in assessing the impact of HIV programs [27, 35, 36]. However, these national surveys take significant amount of time (8–12 months for completion) and resources but detect only few recent infections [51]. Detection of only 10 to 50 recent infections out of 20,000 to 30,000 participants per survey limited our ability to use this information for further sub-group analysis or real-time intervention to prevent ongoing transmission in at risk groups. Estimation of new infections in the U.S. was mainly a retrospective activity and academic in nature [52, 53]. This did not allow timely public health response making a quick impact on ongoing transmission. This may partially explain why we have not seen a significant decline of new infections over the last 15 years in the U.S. Renewed focus on use of the algorithm to facilitate detection of acute infections, contact tracing, and targeted prevention should help prevent ongoing transmission and help reduce new infections [54].

The development of a rapid test for recent infection can quickly identify individuals who are recently infected, as this is the target group that is known to contribute significant transmission of HIV due to their immature immune response and high viral load [17, 24]. Some estimates suggest that 40%-60% of ongoing HIV transmission may be contributed by recently infected persons [44, 45, 55, 56]. Thus, early identification of recent infections, concomitant with HIV treatment and viral suppression, would interrupt HIV transmission at an early stage accelerating epidemic control. Contact tracing and partner testing will inherently increase the yield of HIV-positive cases as well as prevent further transmission through counseling and the provision of PrEP to those at risk. This shift from monitoring only cross-sectional populations to identifying new infections in the routine HIV testing services (HTS) and most importantly at the POC, will serve as an important epidemic control tool to reach zero new infections.

We used the Asante strip reader in conjunction with visual reading to evaluate its performance and establish reader cutoffs becau**se** it is useful in order to ensure kit lot consistency. The Asante strip reader is relatively expensive and may not be practical to use at the testing sites. Our results show that there is a high level of agreement between visual and strip reader results for both the PVL and LT lines (99.87% and 98.05% respectively). A few specimens changed classification on both reads, but these were limited to samples that were borderline which is expected. Based on the comparative results, a strip reader is not needed for the identification of recent infection for the intended use in public health surveillance as visual results are substantially similar to strip reader results. Because of this and other issues such as annual maintenance, calibration, fragility, and difficulty of using the reader at POC, we do not currently recommend the use of a strip reader at site level where rapid tests are routinely conducted.

As part of limitations, we do recognize that the ARRA is an antibody-based test similar to LAg-Avidity EIA and, therefore, will require use of the appropriate recent infection testing algorithm (RITA) to include viral load to correctly classify recent infection cases [27, 57]. If implemented in conjunction with case-based surveillance, it is possible that unique ID and clinical history can identify retesting individuals and individuals on ART, thus minimizing the need for VL. We also acknowledge the need for additional studies to determine and confirm MDRI using seroconversion panels and using specimens with varying duration of treatment, including breakthrough infections among those on pre-exposure prophylaxis (PrEP). Future studies using specimens with additional demographic and HIV-1 subtype information can be helpful to better understand performance and limitations of the assay. Additional limitation is HIV-2 positive specimens which, in the absence of type-specific diagnosis, will be classified as recent infections since rIDR-M antigen used for recency classification is derived from HIV-1 gp41 immunodominant region [20] and will not react with HIV-2 specimens. If HIV-2 is suspected, type-specific diagnosis should be conducted using peptide-based assays such as Multispot, Geenius or InnoLIA.

RTRI is now being implemented in several PEPFAR-supported countries as a key tool to accelerate epidemic control [58–60]. Although our study and additional data from other laboratories demonstrate high sensitivity and specificity of ARRA for HIV diagnosis, the test is not currently used as part of the national testing algorithm for HIV diagnosis because of lack of regulatory approval. Accordingly, the test is used as an addition to the national testing algorithm which increases the cost of testing and labor. Historically, HIV-1 incidence assays have been used mainly for incidence surveillance and did not require regulatory approval. However, future regulatory approval of a RTRI that combines HIV diagnosis and recency status in the same device will be beneficial to further simplify HIV diagnosis as well as recent infection surveillance.

## Supporting information

S1 Data Excel file with raw data that was used for all analysis shown in the paper. (XLSX)
