# Correction: Performance evaluation of the Asante Rapid Recency Assay for verification of HIV diagnosis and detection of recent HIV-1 infections: Implications for epidemic control

**DOI:** 10.1371/journal.pgph.0002287

**Published:** 2023-08-04

**Authors:** Ernest L. Yufenyuy, Mervi Detorio, Trudy Dobbs, Hetal K. Patel, Keisha Jackson, Shanmugam Vedapuri, Bharat S. Parekh

There are errors in the article Abstract. The correct Abstract is: We previously described development of a rapid test for recent infection (RTRI) that can diagnose HIV infection and detect HIV-1 recent infections in a single device. This technology was transferred to a commercial partner as Asante Rapid Recency Assay (ARRA). We evaluated performance of the ARRA kits in the laboratory using a well-characterized panel of specimens. The plasma specimen panel (N = 1500) included HIV-1 (N = 570), HIV-2 (N = 10), and HIV-negatives (N = 920) representing multiple subtypes and geographic locations. Reference diagnostic data were generated using the Bio-Rad HIV-1-2-O EIA/Western blot algorithm with further serotyping performed using the Multispot HIV-1/2 assay. The LAg-Avidity EIA was used to generate reference data on recent and long-term infection for HIV-1 positive specimens at a normalized optical density (ODn) cutoff of 2.0 corresponding to a mean duration of about 6 months. All specimens were tested with ARRA according to the manufacturer’s recommendations. Test strips were also read for line intensities using a reader and results were correlated with visual interpretation. ARRA’s positive verification line (PVL) correctly classified 575 of 580 HIV-positive and 910 of 920 negative specimens resulting in a sensitivity of 99.1% (95% CI: 98.0–99.6) and specificity of 98.9% (95% CI: 98.1–99.4), respectively. The reader-based classification was similar for PVL with sensitivity of 99.3% (576/580) and specificity of 98.8% (909/920). ARRA’s long-term line (LTL) classified 109 of 565 HIV-1 specimens as recent and 456 as long-term compared to 98 as recent and 467 as long-term (LT) by LAg-Avidity EIA (cutoff ODn = 2.0), suggesting a mean duration of recent infection (MDRI) close to 6 months. Agreement of ARRA with LAg recent cases was 81.6% (80/98) and LT cases was 93.8% (438/467), with an overall agreement of 91.7% (kappa = 0.72). The reader (cutoff 2.9) classified 109/566 specimens as recent infections compared to 99 by the LAg-Avidity EIA for recency agreement of 81.8% (81/99), LT agreement of 94% (439/467) with overall agreement of 91.9% (kappa = 0.72). The agreement between visual interpretation and strip reader was 99.9% (95% CI: 99.6–99.9) for the PVL and 98.1% (95% CI: 96.6–98.9) for the LTL. ARRA performed well with HIV diagnostic sensitivity >99% and specificity >98%. Its ability to identify recent infections is comparable to the LAg-Avidity EIA corresponding to an MDRI of about 6 months. This point-of-care assay has implications for real-time surveillance of new infections among newly diagnosed individuals for targeted prevention and interrupting ongoing transmission thus accelerating epidemic control.
